# Comparison of different scales for the evaluation of anxiety and compliance with anesthetic induction in children undergoing scheduled major outpatient surgery

**DOI:** 10.1186/s13741-021-00228-x

**Published:** 2021-12-14

**Authors:** Alberto Vieco-García, Amanda López-Picado, Manuel Fuentes, Laura Francisco-González, Belén Joyanes, Carmen Soto, Ana Garcia de la Aldea, Carlos Gonzalez-Perrino, Esther Aleo

**Affiliations:** 1 Unidad de Cuidados Intensivos Pediátricos y Unidad de Recuperación Postanestésica, Servicio de Pediatría (6° planta sur), Hospital Clínico San Carlos, C/Profesor Martin Lagos s/n, 28040 Madrid, Spain; 2 Departamento de Pediatría y Neuropediatría, Clínica Universidad de Navarra, Campus Madrid, Madrid, Spain; 3 Unidad de Investigación Clínica y Ensayos Clínicos, Hospital Clínico San Carlos, Instituto de Investigación Sanitaria del Hospital Clínico San Carlos (IdISSC), Madrid, Spain; 4 Facultad de Salud, Universidad Internacional de La Rioja, Logroño, Spain; 5 Unidad de apoyo metodológico a la Investigación, Hospital Clínico San Carlos, Instituto de Investigación Sanitaria del Hospital Clínico San Carlos (IdISSC), Madrid, Spain; 6 Cirugía Pediátrica, Hospital Clínico San Carlos, Madrid, Spain; 7 Servicio de Anestesiología, Hospital Clínico San Carlos, Madrid, Spain; 8 Departamento de Pediatría, Facultad de Medicina, Universidad Complutense de Madrid, Madrid, Spain

**Keywords:** Perioperative anxiety, Scales, Separation from the parents, Anesthetic induction

## Abstract

**Introduction:**

Anxiety in children triggered by a scheduled surgical intervention is a major issue due to its frequency and consequences. Preoperative anxiety is associated with increased patient fear and agitation on anesthetic induction. The aim of this study is to compare three preoperative anxiety scales for children undergoing elective outpatient surgery, and to correlate each of these tools with the degree of patient compliance on induction, as assessed by the Induction Compliance Checklist (ICC).

**Methods:**

An observational prospective study was performed on a cohort of children with ages between 2 and 16 years old, scheduled for outpatient surgery. Anxiety was assessed upon arrival to the hospital (M0), during transfer to the surgical unit (M1), and in the operating room during anesthetic induction (M2). Anxiety in the parents (measured with the State-Trait Anxiety Inventory, STAI) and in the children (measured with the Spence Anxiety Scale-Pediatric, SCAS-P, the State-Trait Anxiety Inventory Children, STAIC, and Modified Yale Preoperative Anxiety Scale, m-YPAS) was assessed. Compliance with anesthetic induction was assessed with ICC.

**Results:**

The study included 76 patients (72.4% male, median age 7.9 years). Anxiety scores (m-YPAS) increased as the moment of surgery approached, being greater at the entrance to the surgical unit (M0 = 26.1 ± 9.5; M1 = 31.8 ± 18.1; M2 = 33.5 ± 21.1). A strong correlation was found between ICC scale and m-YPAS at M1 (0.738) and M2 timepoints (0.794), but not with the rest of scales at M0.

**Conclusions:**

Standard anxiety assessment scales do not predict the quality of anesthetic induction. m-YPAS scale can detect increasing anxiety in children as they approach the surgical procedure and this correlates strongly with a worse anesthetic induction, defined by higher score on ICC scale.

**Supplementary Information:**

The online version contains supplementary material available at 10.1186/s13741-021-00228-x.

## Introduction

Scheduled major outpatient surgeries, such as phimosis, inguinal hernia, or cysts, are the most frequent surgical procedures performed in children (Observatorio de resultados en salud de la Comunidad de Madrid-Atencion Especializada 2019). Surgical procedures can generate significant preoperative anxiety (POA) in as much as 70% of the pediatric population. This is mainly due to separation from their parents when faced with the threat posed by a strange environment and the uncertainty about the procedure, as well as the potential suffering it may entail. In addition, the non-urgent nature of a scheduled surgical procedure can contribute to increase POA as some children may perceive it as a punishment since they feel otherwise well (Garanhani and Valle 2012). Other factors that may also be related to POA include age, temperament, behavioral problems, prior surgeries and hospitalizations, and parental anxiety (Garanhani and Valle 2012; Chorney and Kain 2009; Kain et al. 2006; Hudek 2009; Kain et al. 2004; Deshpande et al. 1993; Kain 1997).

Children with higher POA experience more perioperative complications, such as prolonged anesthetic induction, worse post-anesthetic recovery, need for higher doses of post-surgical analgesia and delirium on awakening (Kain et al. 2006; Lee et al. 2012). Moreover, all these factors translate into an increase in hospital costs and a worse perception of the quality of healthcare (Hudek 2009). In addition, in the mid and long term, children with POA are more likely to develop maladaptive behaviors, such as separation anxiety, general behavioral changes, aggressiveness, eating disorders, insomnia, and nocturnal eneuresis (Kain et al. 2006; Hudek 2009; Kain et al. 2004; Deshpande et al. 1993; Kain 1997; Lee et al. 2012).

The poor quality of anesthetic induction correlates with poorer results during and after surgery (Chorney and Kain 2009; Hudek 2009; Kain et al. 2004). It seems that one of the main factors that contribute to an inadequate anesthetic induction is anxiety and stress upon arrival in the operating room. Anxiety related to the surgical process can be measured by different validated scales. Some are self-questionnaires created to assess anxiety based on the age of the patient, although not in the surgical setting, such as the SCAS-P and the STAI-C. The m-YPAS scale has been designed to measure anxiety in this context and it is validated in children of 2-12 years.

Early identification of those children undergoing SMOS with POA at risk of developing a poor anesthetic induction would allow on time interventions that could potentially translate into improved perioperative outcomes. This could also result in reduced costs as well as improved patient and caregivers’ satisfaction. It is therefore important to identify the most appropriate assessment tool for this purpose in this particular setting.

In the present study, we compare three different scales for the assessment of presurgical anxiety in children undergoing SMOS at different time points in their perioperative journey.

Our objectives are to determine which is the best tool for the assessment of POA in this context and to investigate the link between POA and the quality of anesthetic induction. Our hypothesis is that POA has a negative impact on the quality of anesthetic induction and this can be detected before entering the operating room.

## Methods

The study was reviewed and approved by the Clinical Research Ethics Committee of the Hospital Clínico San Carlos.

This is a longitudinal prospective observational study of a cohort of pediatric patients who underwent a SMOS procedure at a tertiary care university hospital, from October 2015 to March 2017. The patients were selected consecutively from the surgical outpatient clinic and enrolled in the study if they fulfilled the inclusion-exclusion criteria.

O *Inclusion criteria*: Patients between the ages of 2 and 16 years who were scheduled for SMOS by the Pediatric Surgery Department, with an anesthetic risk assessment ASA I or II. Informed signed consent was obtained from the parents or legal guardians, as well as the children consent if aged 12 or older, before inclusion in the study.

O *Exclusion criteria*. Patients older than 2 years of age with a history of prior surgery. Children with a history of previous surgeries before this age were considered not to retain any memories of the surgical experience and therefore, this was not considered an exclusion criterion.

### Variables analyzed

The collection of data for the study was limited to the day of the intervention, from admission to the time of anesthetic induction.

- Epidemiological data collected included age, sex, and the surgical procedure to be performed.
- Anxiety scales not specific to the surgical procedure. These are validated self-reported questionnaires to assess state-trait anxiety. They were completed by the family upon arrival at the surgical day hospital (M0).
  - ◦ Spence Anxiety Scale-Pediatric (SCAS-P) (Hernández-Guzman et al. 2010). This is a validated scale to measure anxiety in children between 2 and 5 years of age through the parent’s direct responses. The higher scores reflect a greater degree of anxiety.
  - ◦ State-Trait Anxiety Inventory Children (STAIC) (Spielberger 1973). This scale evaluates anxiety in children between 5 and 16 years of age. It assesses 20 items of the anxiety state and 20 items of the anxiety trait (proneness to perceive anxiety), with scores between 1 and 3 points depending on the degree of anxiety, ranging from 20 (low anxiety) to 60 points (maximum anxiety). The final interpretation is based on the percentiles and Z-scores according to sex and age. The reliability assessed with the Cronbach alpha coefficient was 0.82 for boys and 0.87 for girls, implying an acceptable internal consistency of the scale to measure anxiety.
  - ◦ State-Trait Anxiety Inventory (STAI). This scale was completed by the child’s father or mother (at their choice). It assesses parental anxiety in state and trait anxiety blocks, each with 20 items. The scores vary from 0 to 60 and the scale’s scores are interpreted according to their conversion into the relevant percentiles and on a scale of 10. The test re-test has a high correlation (0.73-0.86), and its validity has been demonstrated with high and low levels of anxiety in studies performed on large cohorts of students. The correlation values obtained of 0.83 to 0.94 suggest a very strong validity (Jerez et al. 2016b).
- Scales specific to the surgical context
  - ◦ Modified Yale Preoperative Anxiety Scale (m-YPAS) (Kain et al. 1995). This scale is validated (Jerez et al. 2016a) for its use in children between 2 and 12 years of age. Its extended version consists of 22 items, grouped into 5 domains: activities, vocalization, emotional expressivity, state of awareness, and interaction with family members. In this scale, higher scores reflect a higher level of anxiety. The short version comprises 18 items, and does not consider the interaction with family members, with a minimum score of 23 and a maximum score of 100 (Jerez et al. 2016b). With both scales, the presence of anxiety is defined by a score greater than or equal to 30. The evaluation of the m-YPAS scales at each of the three time points was performed by the same observer.
  - ◦ Induction Compliance Checklist (ICC) (Kain et al. 1998). This is an observational scale to measure the cooperation and behavior of the patient during anesthetic induction. It uses a checklist of 10 items that correspond to negative behaviors that are frequently observed at such time, scoring 1 point when these are present and 0 points if these are not observed (see supplemental material). The ICC score is obtained by summing the scores for all the items (10 points maximum). Perfect anesthetic induction (with no negative behaviors) scores 0 points, suboptimal induction is scored between 1 and 4 and a score above 4 is considered as poor induction. The ICC scale has only mild inter-observer and intra-observer variability (r > 0.995).

### Study procedure

After the informed consent was signed, the patient’s POA was assessed at the following points during the day of the intervention.

#### Time point 0 (M0)

This is the time when the patient arrived at the pre-operative hospitalization area. At this point, the researchers recorded a video to assess the patient’s initial state of anxiety using the m-YPAS (extended version). The self-reported questionnaires of the non-specific anxiety scales (STAI, SCAS-P, or STAIC) were completed by the child or the parents as appropriate and based on the patient’s age.

#### Time point 1 (M1)

The moment when the patient is transferred to the surgical area, at the entry of the operating theater, and he/she is separated from the parents. The researchers again recorded a video at this timepoint to assess later the subject’s preoperative anxiety using the m-YPAS scale (extended version).

#### Time point 2 (M2)

This is the moment of anesthetic induction in the operating theater. The researchers also recorded a video at this time point to assess the subject’s preoperative anxiety using the m-YPAS scale (short version) and the anesthesiologist completed the ICC.

### Statistical analysis

Qualitative variables are presented as frequency distributions, while quantitative variables are summarized as the means and standard deviations (SD), or the median and interquartile range (IQR) according to presence or absence of normality of the distribution of each variable.

The Student’s *T* test was used to compare means between two groups, while the linear relationship between quantitative variables was evaluated by calculating the Pearson correlation coefficient (*r*). To assess the predictive capacity of the different scales to differentiate patients with difficulties when anesthesia is induced, the area under the curve (AUC) and its 95% CI was calculated. In all hypothesis testing, the null hypothesis was rejected with a type I error or error *α* < 0.05. All the statistical analyses were performed using the statistical package SPSS 15.0.

## Results

Inter-observer reliability in the assessment of the m-YPAS scale with the video recordings was analyzed in a group of 30 subjects at the three times points before the initiation of the study. The intraclass correlation coefficient between the 2 observers was calculated to assess the inter-observer reliability, and the reliability proved to be very good, with an intraclass correlation coefficient of 0.949 at M0 (95% confidence interval CI, 0.895-0.975); 0.800 at M1 (95% CI, 0.607-0.904); and 0.838 at M2 (95% CI, 0.682-0.922).

Seventy-six patients (72.4% male) were included in this study, 23 (30.3%) between 2 and 5 years of age, 35 (46.1%) between 5 and 12 years, and 18 (23.7%) between 12 and 16 years. The characteristics of the children included are summarized in Table 1, and the assessment of anxiety using the m-YPAS scale is shown in Table 2. Considering a cut-off value of 30, the percentage of children who presented anxiety at M0 was 10.5%. This proportion increased to 26.3% when the children were transferred to the surgical unit (M1). Although at the time of anesthetic induction (M2) the proportion of children with anxiety was lower (25%), the mean intensity of anxiety observed according to the m-YPAS scale was the highest of the three moments analyzed (33.5 ± 21.1).

**Table 1:** Characteristics of the patients included in the study

		*n* (%)
Age (years)	Median (IQR) 7.3 (4.3-12.1)	
Sex	Male	55 (72.4)
	Female	21 (27.6)
Surgery	Phimosis	32 (42.1)
	Inguinal hernia	19 (25.0)
	Cysts, fistulas and skin lesions	18 (22.4)
	Other surgeries	6 (7.9)
	Double surgical interventions	2 (2.6)

*IQR* Interquartile range

**Table 2:** Results of preoperative anxiety evaluations in parents and patients performed with specific and non-specific scales of the surgical context

SCALE		***n***	MEAN SCORE (SD)
**m-YPAS 0**		**76**	**26.1 (9.5)**
**m-YPAS 1**		**76**	**31.8 (18.1)**
**m-YPAS 2**		**76**	**33.5 (21.1)**
***SCAS-P***		**25**	**18.9 (10.8)**
***STAIC***	***STAIC-S***	**49**	**32.8 (7.4)**
	***STAIC-T***	**49**	**32.3 (6.8)**
***STAI***	***STAI-S***	**76**	**19.7 (9.7)**
	***STAI-T***	**76**	**18.6 (9.6)**

*n* number of performed evaluations, *SD* Standard deviation, *m-YPAS* modified Yale preoperative anxiety scale, *m-YPAS 0* m-YPAS in moment 0 or arrival at the hospital, *m-YPAS 1* m-YPAS in moment 1 or during transfer to the surgical unit, *m-YPAS 2* m-YPAS in moment 2 or arrival at the operating theater for anesthetic induction, *SCAS-P* Spence Anxiety Scale-Pediatric, *STAIC* State-Trait Anxiety Inventory-Children, *STAIC-S* STAIC-State anxiety, *STAIC-T* STAIC-Trait anxiety, *STAI* State-Trait Anxiety Inventory, *STAI-S* STAI-State anxiety, *STAI-T* STAI-Trait anxiety

The results of SCAS-P, STAIC, and STAI scales, which are not specific for the surgical context, did not demonstrate the presence of anxiety in the population analyzed (Table 2), when compared to m-YPAS at the same time point (M0) (Table 3). By contrast, at M0, the STAIC-T scale showed significantly higher scores in children of 5 to 16 years of age with anxiety, according to the results of m-YPAS, (M0, 42.2 ± 7.6) compared with those without anxiety (31.5 ± 6.1; *p* = 0.002). Moreover, a positive correlation was found between m-YPAS (M0) and STAIC-T (*r* = 0.441, *p* = 0.002). No significant differences were obtained for the rest of the scales.

**Table 3:** Average score of each scale based on the presence or absence of anxiety according to the m-YPAS scale at moment 0

		m-YPAS 0 < 30 (no anxiety)		m-YPAS 0 > 30 (anxiety)		
SCALE		***n***	mean (SD)	***N***	mean (SD)	***p***
	**SCAS-P**	21	18.5 (9.6)	4	20.7 (17.6)	0.824
**STAIC**	**STAIC-S**	45	32.4 (7.2)	4	37.2 (9.3)	0.222
	**STAIC-T**	**45**	**31.5 (6.1)**	**4**	**42.2 (7.6)**	**0.002**
**STAI**	**STAI-S**	68	19.9 (9.5)	8	18.2 (11.5)	0.645
	**STAI-T**	68	18.7 (9.9)	8	17.7 (7.2)	0.793

*SD* Standard deviation, *m-YPAS* modified Yale preoperative anxiety scale, *m-YPAS 0* m-YPAS in moment 0, *SCAS-P* Spence Anxiety Scale—Pediatric, *STAIC* State-Trait Anxiety Inventory Children, *STAIC-S* STAIC-State anxiety, *STAIC-T* STAIC-Trait anxiety, *STAI* State-Trait Anxiety Inventory, *STAI-S* STAI-State anxiety, *STAI-T* STAI-Trait anxiety

A linear correlation analysis was performed between the non-specific scales. A significant mild-moderate correlation was observed between parental (both trait and state anxiety) and children’s anxiety, although only for those younger than 5 years of age (Table 4).

**Table 4:** Contingency study between self-administered scales non-specific to the surgical context

Pearson correlation, r (p)	SCAS-P	STAIC-T	STAIC-S	STAI-S
**STAI-T**	**0.385 (0.057)**	0.221 (0.128)	−0.081 (0.580)	**0.608 (< 0.001)**
**STAI-S**	**0.416 (0.038)**	0.107 (0.466)	0.163 (0.262)	1
**STAIC-T**	NA	1	0.190 (0.190)	0.107 (0.466)
**STAIC-S**	NA	0.190 (0.190)	1	0.163 (0.262)

*r* Pearson correlation coefficient, *SCAS-P* Spence Anxiety Scale-Pediatric, *STAIC* State-Trait Anxiety Inventory—Children, *STAI* State-Trait Anxiety Inventory, *STAI-S* STAI-State anxiety, *STAI-T* STAI-Trait anxiety, *STAIC-S* STAIC-State anxiety, *STAIC-T* STAIC-Trait anxiety, *NA* Not applicable

The quality of anesthetic induction was analyzed using the ICC scale. In total, 76.3% (*n* = 58) of the cohort experienced a perfect induction (ICC = 0, absence of anxiety), whereas in 23.6% (*n* = 18) induction was sub-optimal or poor. A significant correlation was found between the ICC and m-YPAS scores at M1 (*r* = 0.615; *p* < 0.001) and an even stronger correlation at M2 (*r* = 0.779; *p* < 0.001) (Table 5). These results indicate that a worse anesthetic induction was significantly associated with higher levels of anxiety in our patients. No correlations were found between the non-specific scales and the ICC. We also evaluated the capacity of m-YPAS to predict compliance with the anesthetic induction and significant AUC for ROC (receiver operating characteristics) curves were obtained for M1 and M2 timepoints. In this regard, the predictive capacity of this scale was moderate-high at M1 (0.738; 95% CI = 0.598-0.877) and high at M2 (0.794; 95% CI = 0.600-0.937).

**Table 5:** Comparison between the mean values obtained from the specific and non-specific scales of the surgical context and the ICC scale. Correlation study between both scales and the ability of the m-YPAS scale to predict adequate induction compliance

			Perfect induction (ICC = 0)		Imperfect induction (ICC > 0)		***p***	Pearson correlation, r (***p***)	ROC curves (IC 95%)
			Mean score (SD)	***n***	Mean score (SD)	***N***			
**Specific and non-specific scales of the surgical context**	**m-YPAS**	**m-YPAS 0**	26.2 (10)	58	25.9 (8.2)	18	0.917	−0.095 (0.415)	0.474 (0.322-0.626)
		**m-YPAS 1**	28.0 (10.7)	58	44.2 (29.3)	18	**0.001**	**0.615 (< 0.001)**	**0.738 (0.598-0.877)**
		**m-YPAS 2**	26.4 (10.8)	58	56.5 (28.9)	18	**< 0.001**	**0.779 (< 0.001)**	**0.794 (0.6-0.937)**
	**SCAS-P**		18.7 (10.5)	13	19.0 (11.6)	12	0.944	0.049 (0.816)	0.487 (0.253-0.722)
	**STAIC**	**STAIC-S**	32.5 (7.5)	44	36.0 (6.2)	5	0.324	0.174 (0.233)	0.661 (0.446-0.877)
		**STAIC-T**	32.2 (6.8)	44	33.8 (7.8)	5	0.633	0.038 (0.795)	0.539 (0.241-0.836)
	**STAI**	**STAI-S**	19.5 (10.0)	58	20.3 (8.9)	18	0.778	0.018 (0.879)	0.523 (0.372-0.673)
		**STAI-T**	18.7 (9.2)	58	18.0 (11.1)	18	0.784	−0.009 (0.938)	0.450 (0.277-0.623)

*ICC 0* perfect induction, *ICC > 0* imperfect induction, *X* mean, *SD* standard deviation, Pearson correlation analysis and Receiver Operating Characteristic (ROC) curves, *m-YPAS* modified Yale preoperative anxiety scale, *SCAS-P* Spence Anxiety Scale—Pediatric, *STAIC* State-Trait Anxiety Inventory—Children, *STAI* State-Trait Anxiety Inventory, *STAI-S* STAI-State anxiety, *STAI-T* STAI-Trait anxiety, *STAIC-S* STAIC-State anxiety, *STAIC-T* STAIC-Trait anxiety

The same analysis was done for the specific population of adolescents of 12-16 years of age. ROC curves were generated for the correlation between the ICC and the m-YPAS at M1, obtaining an AUC of 0.875 (95% CI = 0.713-1.00, high predictive capacity), and with the m-YPAS at M2, obtaining an AUC of 0.703 (medium-high predictive capacity) for this age group.

## Discussion

The results of this study demonstrate that POA increases along the preoperative journey and that higher levels of anxiety are associated with worse anesthetic induction experiences in children undergoing SMOS. In this regard, our results suggest that m-YPAS is the most adequate scale to assess the evolution of POA in this context and that it has a strong capacity to predict the results of anesthetic induction, as opposed to other non-specific anxiety scales.

Our cohort is one of the largest in pediatric studies on POA published to date (Hudek 2009; Fincher et al. 2012; Kain et al. 1998), and to our knowledge, this is the first study to compare different scales for the evaluation POA. Regardless of the scale used, low levels of anxiety were observed in all patients upon arrival at the hospital (M0). However, the children’s anxiety increased as they progressed through the preoperative journey to the operating theater. The separation from their parents was the moment when most patients experienced POA, although the highest anxiety scores were observed at the time of anesthetic induction. Previous studies have also indicated that anesthetic induction is moment of maximal anxiety [4, 14, 22]. The lack of coincidence between the moment with the highest proportion of anxious patients and that with the highest POA scores in our study can be explained by the fact that in our hospital the children are separated from their parents when they enter the operating theater, whereas in most previous studies the parents remain with their children until the moment of anesthetic induction (Kain et al. 2006; Hudek 2009; Sadeghi et al. 2016).

Parental presence during anesthetic induction is one of the best studied non-pharmacological interventions to reduce POA. As a consequence, this strategy has been implemented in many countries with little negative impact (Kain et al. 2006; Hudek 2009), 26]. However, there are conflicting results regarding the impact of parental presence on surgical outcomes (Yip et al. 2009; Sadhasivam 2010; Vagnoli et al. 2010), 26].

According to our data, children appeared to experience more trait and state anxiety than their parents, although within the average levels of anxiety for this population. We found congruent correlations between state and trait anxiety measured with the self-questionnaire of the STAI scale in parents, i.e., the subjects with more anxious traits had higher levels of state anxiety. Furthermore, we observed a significant correlation between evaluation of anxiety in children under 5 years of age (evaluated with SCAS-P) and their parents, both in state and trait anxiety (STAI S/T). The SCAS-P scale was completed by the parents of children aged 2-5 years and we found a significant correlation between the SCAS-P scale and the STAI-S or STAI-T scales, so that when the SCAS-P score increased so did the STAI-S and STAI-T scores. This may be explained by the fact that both scales were completed by the child’s mother or father. In fact, a positive correlation between children’s anxiety and parental anxiety has been described previously (Kain et al. 1998). Therefore, according to our study, parents’ anxiety (both trait and state anxiety) appears to influence their children’s anxiety, only in those under 5 years of age.

Regarding the possibility of predicting POA, we found a significant correlation between m-YPAS at M0 and STAIC-T. Consequently, the vital anxiety of children between 5 and 16 years of age may predict the level of POA that they will experience upon arrival at the hospital, i.e., children over 5 years who are more anxious by nature will become anxious more easily when arriving at the hospital. This finding also translates into a concurrent validity between the STAIC-T and the specific m-YPAS scale administered at that same time point (M0). However, none of the self-administered scales that are not specific to the surgical context showed predictive validity with the m-YPAS scale as the patients progressed through the different preoperative stages (M1 and M2), or during anesthetic induction (ICC).

In this study, the moment of greatest anxiety is the closest to the anesthetic induction. Thus, we cannot predict the anxiety that patients will experience by using the self-administered scales upon their arrival at the hospital on the day of surgery. It can be speculated that if these scales were assessed before admission, as reported in previous studies, their predictive capacity would be even lower (Fincher et al. 2012). In this regard, we found a significant positive correlation of the m-YPAS scale with the ICC, which was moderate at M1 and strong at M2. Hence, this scale enables the prediction of a poor anesthetic induction, a prediction that is more reliable; the closer to the moment of anesthetic induction, the m-YPAS scale is administered. This predictive capacity was not found for the non-specific scales.

To our knowledge, this study is the only one to date that has assessed adolescents between 12 and 16 years of age in this context. The pattern of anxiety in this group was similar to that of other age ranges. Likewise, higher levels of anxiety were observed with the m-YPAS at M2 in those subjects who experienced inadequate anesthetic induction. Similarly, the results of the m-YPAS scale at timepoints M1 and M2 were predictive of the quality of anesthetic induction in this age group.

This study has some limitations that must be considered. Firstly, girls are underrepresented in our study. Preoperative behavior and anxiety in girls may be different, as is the bond with their parents. However, the predominance of males in the cohort reflects the high prevalence of phimosis surgery in the study population, probably reflecting the real-world population of pediatric SMOS. Besides, ethnicity of children included in the study was not registered so we have not investigated the influence of this factor on POA. Another limitation of the study is that this population is quite homogeneous. Inclusion criteria of anesthetic risk assessment ASA I or II selected a cohort of relatively healthy children undergoing SMOS with a very limited variety of procedures, and therefore we could not confirm our results in sicker children or in more complex surgeries. Considering all these factors, the extrapolation of our results to other populations should be done with caution. The use of video recording, and in particular, the presence of a person making the video recording may modify the patient’s normal behavior, as proposed previously. However, interaction between the cameraman and the child was avoided in our study to minimize this risk. On the other hand, the large sample size and the inclusion of a group of adolescents between 12 and 16 years of age, who had not been studied previously in this context, are among the strengths of our study.

## Conclusions

In this study, we found a progressive increase in POA related to SMOS from the time of admission to the moment of anesthetic induction among children and adolescents. The information obtained with scales that are not specific to the surgical context are consistent with that obtained with the m-YPAS scale when applied at the same time, yet they do not show any predictive value regarding the subsequent progression of anxiety. When administered at the entry to the surgical unit, the m-YPAS scale can predict the quality of anesthetic induction. Therefore, our results suggest that the m-YPAS scale should be used in children and adolescents at the time of transfer to the surgical unit in order to anticipate the need for therapeutic measures to reduce anxiety at such time, particularly as adequate management of POA could improve the quality of anesthetic induction and ultimately the post-surgical evolution of these patients. It remains to be determined whether the presence of the parents during anesthetic induction influences their child’s anxiety, regardless of previous anxiety state.

## Supplementary Information

**Additional file 1.** Induction Compliance Checklist.

## Data Availability

Information available under request.

## Abbreviations

AUC: Area under the curve
CI: Confidence interval
ICC: Induction Compliance Checklist
IQR: Interquartile range
M0: Time point 0
M1: Time point 1
M2: Time point 2
m-YPAS: Modified Yale Preoperative Anxiety Scale
OSS: Outpatient scheduled surgery
POA: Preoperative anxiety
ROC: Receiver operating characteristics
SCAS-P: Spence Anxiety Scale-Pediatric
SD: Standard deviations
SMOS: Scheduled major outpatient surgery
STAI: State-Trait Anxiety Inventory
STAIC: State-Trait Anxiety Inventory Children
