# Supplementary material for: Comparison of different scales for the evaluation of anxiety and compliance with anesthetic induction in children undergoing scheduled major outpatient surgery
Source: Perioper Med (Lond). 2021 Dec 14;10:58. doi: 10.1186/s13741-021-00228-x (PMC8670148; doi:10.1186/s13741-021-00228-x)
Supplement: Supplementary file 1 — Additional file 1. Induction Compliance Checklist. [file 13741_2021_228_MOESM1_ESM.docx]

**SUPPLEMENTAL MATERIAL**

Supplemental material

Induction Compliance Checklist

| BEHAVIOR | SCORE: YES =1; NO = 0 |
| --- | --- |
| Crying, tears in eyes |  |
| Turns head away from mask |  |
| Verbal refusal, says “no” |  |
| Verbalization indicating fear or worry (where`s mommy? Or will it hurt?) |  |
| Pushes mask away with hands, or pushes nurse/ anesthetist with feet or hands |  |
| Covers motuh/nose with arms/hands, or buries face |  |
| Hysterical crying, may scream |  |
| Kicks, flails legs/arms, arches back, and/or general struggling |  |
| Requires physical restraint |  |
| Complete passivity, either rigid or limp |  |

Total score = the number of categories checked. Perfect induction total score = 0

^6^ Kain Z, Mayers L, Wang S, Caramico L, Hofstadter M. Parental presence during induction of anesthesia versus sedative premedication: which intervention is more effective? Anesthesiol. 1998; 89:1147-56
